# Ontogenetic Changes in Intrinsic Hand and Foot Proportions in Chimpanzees, *Pan troglodytes*

**DOI:** 10.1002/ajpa.70346

**Published:** 2026-09-03

**Authors:** Tom Combemorel, Jesse W. Young, François Druelle

**Affiliations:** ^1^ UMR 7194, Histoire Naturelle Des Humanités Préhistoriques, MNHN, CNRS, UPVD Paris France; ^2^ Department of Biomedical Sciences Northeast Ohio Medical University Rootstown Ohio USA; ^3^ UMR 7268, Anthropologie Bio‐Culturelle, Droit, Ethique et Santé, Aix‐Marseille Université, CNRS, EFS Marseille France; ^4^ Functional Morphology Laboratory University of Antwerp Antwerp Belgium

**Keywords:** evolution, grasping, great ape, ontogeny, phalangeal index

## Abstract

**Objectives:**

Ontogenetic trajectories in intrinsic hand and foot proportions are likely to vary in primates according to their locomotor adaptations. We analyzed the developmental trajectories of chimpanzees ( *Pan troglodytes* ), which exhibit particular locomotor adaptations related to suspensory and climbing abilities, testing whether autopodial growth reflects biomechanical and ecological constraints associated with ontogenetic changes in locomotor behavior.

**Materials and Methods:**

We analyzed 157 radiographs of 7 female and 198 of 8 male *P. troglodytes* individuals, from 1.5 to 13 years old. Lengths of metapodials and proximal/intermediate phalanges were measured to compute phalangeal indices (PIs: ratio of summed non‐distal phalangeal length to corresponding metapodial length). Ontogenetic allometry was assessed by regressing bone length against body mass, with sex included as a factor. Non‐linear Gompertz growth models were fitted to estimate bone‐specific growth rates and durations.

**Results:**

The results show a positively allometric relationship between all bone lengths and body mass. However, sex‐related differences are observed in the allometry of phalanges and metapodials. PIs decline during growth for all digits, with this ontogenetic decline remaining highly similar between sexes. Gompertz models further indicate distinct growth rates between manual and pedal elements.

**Discussion:**

Ontogenetic changes in chimpanzees indicate canalized autopodial growth, with reduced intrinsic proportional changes at ~5 years, aligning with their major positional shift. Comparative analyses show species‐specific phalangeal trajectories, possibly more pronounced in the foot than in the hand and likely reflecting adaptations to ecological and locomotor demands, such as terrestrial knuckle‐walking in large‐bodied chimpanzees.

## 1 Introduction

In primates, juveniles and adults often share the same ecological niche, but juveniles face disadvantages due to their small body size, immature musculoskeletal systems, and lack of experience (Carrier 1996). These constraints increase mortality rates, subjecting young individuals to strong selective pressures that favor anatomical and behavioral adaptations enhancing early‐life survival (e.g., Herrel and Gibb 2006; Hurov 1991; Young and Shapiro 2018; Young et al. 2010; Young 2023). One such adaptive pattern is ontogenetic allometry, where certain structures develop disproportionately to improve functional performance at early stages (Gould 1966; Thompson 1942). For example, juveniles often exhibit greater mechanical advantage in their musculoskeletal systems, enabling higher relative force output, as well as increased skeletal robustness to resist fractures during locomotor maturation (Carrier and Leon 1990; Carrier 1996; Heinrich et al. 1999; Kilbourne and Makovicky 2012; Ruff 2003; Young et al. 2010). These selective pressures are particularly pronounced in primates, especially great apes, due to their slow postnatal growth rates, which extend the period of juvenile vulnerability (Ross 1998; Leigh 2001). During the transition to autonomous locomotion, juveniles display higher frequencies of grasping behaviors, such as climbing and suspensory behaviors, compared to adults (e.g., baboons: Druelle et al. 2016; chimpanzees: Doran 1992; Sarringhaus et al. 2014; colobus monkeys: Dunham 2015; gorillas: Doran 1997; macaques: Wells and Turnquist 2001). Morphologically, the larger hands and feet of young primates appear to be an adaptive trait maintained by natural selection (Lawler 2006; Young 2005; Boulinguez‐Ambroise et al. 2021). These anatomical features increase contact surface and grasping capacity, enabling juveniles to use adult‐sized substrates and navigate complex arboreal environments more effectively (e.g., Grand 1977; Preuschoft 2004; Lawler 2006; Druelle et al. 2017; Young and Chadwell 2020). Growth allometry studies, both cross‐sectional and longitudinal, further revealed that distal segments (hands and feet) grow more slowly than proximal segments, resulting in negative allometry for autopodial length (e.g., Lumer and Schultz 1947; Gavan 1951; Grand 1977; Jungers and Fleagle 1980; Ravosa et al. 1993; Turnquist and Wells 1994; Raichlen 2005a; Lawler 2006). This pattern appears to directly influence the way young primates walk (Raichlen 2005b) and move in general (Wells and Turnquist 2001; Lawler 2006; Druelle et al. 2016).

Beyond the relative size of the autopodia, intrinsic proportions (i.e., the length of the free digits relative to the length of the metapodials) are critical for grasping performance, as they improve the ability to fully encircle a greater variety of substrates (e.g., Napier 1967, 1980; Lemelin 1999; Hamrick 2001; Kirk et al. 2008; Sustaita et al. 2013; Druelle et al. 2017; Young and Heard‐Booth 2016; Young et al. 2019; Young 2023). Overall, primates appear to be characterized by short metapodials and long proximal phalanges (Hamrick 2001). Several studies have specifically investigated the functional link between the relative lengths of digits and metapodials and the use of small‐diameter supports and grasping capacities. These can serve as reliable indicators of locomotor specializations in primates (e.g., Lemelin 1999; Druelle et al. 2018; Toussaint et al. 2025). However, few studies have explored whether these intrinsic proportions change during ontogeny. Recent longitudinal studies using periodic radiographs (i.e., on *Cebus* and *Papio*) are among the few datasets that directly address this question. They revealed that the intrinsic proportions of hands and feet also change during ontogeny in primates due to differential allometry in metapodial and phalangeal lengths. In olive baboons, *Papio anubis*, and capuchin monkeys, *Cebus albifrons* and *Sapajus apella*, juveniles have relatively longer digits than adults, supporting the hypothesis that selection favors enhanced grasping ability early in life to facilitate clinging during transport and early arboreal locomotion (Young and Heard‐Booth 2016; Druelle et al. 2017). However, a similar ontogenetic pattern has been observed in a non‐primate animal, the rat ( *Rattus norvegicus* ), challenging the view that these developmental trends are particular to primates (Young et al. 2019). This finding raises the possibility that a general mammalian model of autopodial growth exists, wherein immature animals always have relatively longer digits. Nevertheless, unlike in rats, the positional behavior of adult primates diverges from that of juveniles. Whereas quadrupedalism is characteristic of all infant primates, locomotor modes routinely become more specialized as individuals age, and are thus reflected in particular morphological adaptations of the mature hand and feet (Smith et al. 2020). In this context, the uniqueness of primates may lie not in the developmental pattern of intrinsic proportions itself, but in the magnitude of allometric scaling in metapodials and phalanges and its functional significance for adult locomotion. We hypothesize that this pattern evolved in response to the interaction between body size and species‐specific ecological and locomotor constraints. Studying the developmental trajectory of metapodials and phalanges in species with varying body size and locomotor ecologies, such as chimpanzees ( *Pan troglodytes* ), which are large and heavily reliant on arboreal substrates, could provide valuable insights into how adult locomotor adaptations and ecological demands shape developmental trajectories, filling an important gap in this analytical framework.

The chimpanzee is an ideal model for investigating ontogenetic shifts because it exhibits particularly long digits at the adult stage and a prolonged growth period similar, in some respects, to humans (Hamada et al. 2003; Hamada and Udono 2002). The locomotor repertoire of chimpanzees is unique: despite their large body size, they retain high levels of arboreality, relying on climbing and suspensory behaviors for getting their food (Doran 1992; Sarringhaus et al. 2014; Drummond‐Clarke et al. 2024), while also employing quadrupedal knuckle‐walking on the ground. Crucially, chimpanzees show ontogenetic shifts in locomotor behavior (e.g., Doran 1992; Sarringhaus et al. 2014), which have been linked to the development of specific bony adaptations, such as the dorsal metacarpal ridge which is a plastic feature correlated with increased knuckle‐walking frequency (Sarringhaus et al. 2022). Given their large body mass, arboreal locomotion likely imposes substantial biomechanical constraints, maintaining selective pressure for effective grasping into adulthood (Thompson et al. 2018). Their adult hands and feet, characterized by elongated digits with particularly long metapodials and a relatively short thumb, likely reflect evolutionary trade‐offs related to their unique locomotor development. Studying chimpanzee ontogeny thus provides an ideal framework to test whether the ontogenetic trajectory of autopodial proportions varies with adult locomotor ecology and body size and to understand how ontogenetic patterns shape functional morphology.

Here, we test the hypothesis that the intrinsic hand and foot proportions in chimpanzees change during development and that these changes correspond to the most significant shift in locomotor development, given the size constraints of being large‐bodied in arboreal environments. To do so, we analyzed the ontogeny of intrinsic hand and foot proportions in chimpanzees and how it may be related to functional shifts in locomotor behaviors. We also investigated males and females separately due to sexual dimorphism in chimpanzees. However, despite this dimorphism, we expect intrinsic proportions to remain constant, as differences in body proportions have been shown to result from body size allometry rather than proportional differences as such (Druelle et al. 2017; Huxley 1942; Turnquist and Kessler 1989). Furthermore, we will also specifically discuss our results on chimpanzee ontogeny with published data of the same type in two other primates, capuchins and baboons. Indeed, unlike smaller arboreal primates such as capuchins, and large terrestrial primates such as baboons, chimpanzees are expected to maintain relatively long digits compared to their metapodials, thus strongly influencing the developmental trajectory of this anatomical trait. Note that these species exhibit substantial differences in life history traits, particularly in growth rate and longevity. As a result, chronological age alone may not be a reliable basis for cross‐species comparisons. To address this, we use a relative‐age framework based on growth cessation to standardize developmental timelines: 13 years in chimpanzees (Gavan 1971; Curry et al. 2023), 7 years in baboons (Druelle et al. 2017), and 6.5 years in capuchins (Ausman et al. 1982). We expect chimpanzees to exhibit a relatively slower decline in intrinsic autopodial proportions during growth than baboons and capuchins, retaining more “juvenile‐like” grasping morphologies into adulthood.

## 2 Materials and Methods

### 2.1 Radiographic Sample

The chimpanzee radiographs dataset used in this study originates from the *Infant Studies Program*, established in July 1939 at the Yale Primate Laboratory (Connecticut, USA). It produced a longitudinal series of whole‐body radiographs, from birth to adulthood, in a group of 16 chimpanzees. All the information about the radiographic material has been detailed in Thompson et al. (2020). This longitudinal dataset represents today a valuable and unique source to study the skeletal ontogeny of *Pan troglodytes* , as it is one of the few easily available resources of its kind. During development, a suite of anatomical and physiological measurements was taken at regular intervals. Individuals underwent radiographic imaging monthly during the first 2 years of life, tri‐monthly during years 2–4, semiannually during years 4–7, and yearly thereafter.

The current study included 15 individuals (Spratt, the 16th individual, died after 12 months, hence we did not include it in our dataset). Our sample is composed of 7 females and 8 males. Ideally, we selected radiographs from 1.5 years old (to ensure epiphyseal visibility) to the end of chimpanzee growth, estimated from the literature at 13 years of age (Fragaszy and Bard 1997; Curry et al. 2023). Radiographs were more frequently available during early life, allowing us to capture the rapid developmental changes occurring at an early age. After the age of two years, we generally obtained regular one‐year intervals between each selected radiograph. Some exceptions have been taken when radiographs were not available, or in poor condition, to allow further analysis. Overall, our chimpanzee dataset included 355 radiographs: 177 for the foot and 178 for the hand, covering the age range from 1.5 to 13 years (Table 1).

**TABLE 1:** Radiographic sample analyzed by individual.

Individual	Sex	Age range	Number of foot radiographs	Number of hand radiographs
Alf	Male	3.8–13	11	12
Art	Male	1.5–13	14	14
Banka	Female	1.5–13.1	12	12
Bard	Male	1.5–12	14	14
Falla	Female	1.5–12	11	10
Fanny	Female	1.5–12	12	12
Flora	Female	1.5–13.1	12	12
Jed	Male	1.5–13.1	12	13
Jenny	Female	1.5–13	12	12
Jent	Male	1.5–12.1	12	12
Jojo	Female	1.5–13	10	11
Karla	Female	1.5–8	10	9
Ken	Male	1.5–13	15	15
Scarf	Male	1.5–10	11	11
Web	Male	1.8–11.1	9	9
Total	8 males; 7 females		177	178

### 2.2 Measurement Protocol

**FIGURE 1:**
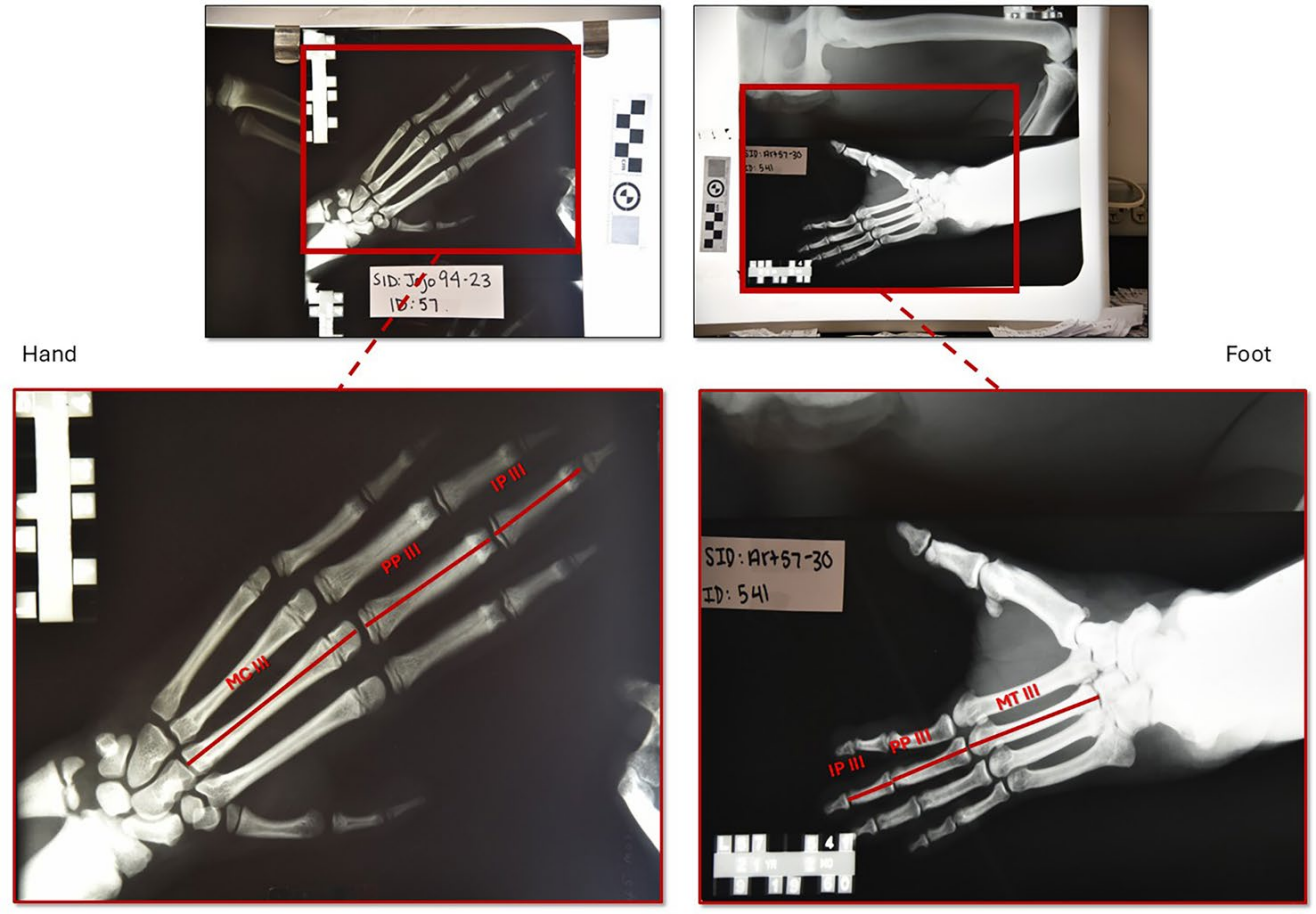
Radiographic measurements of metapodials (MC: Metacarpal, MT: Metatarsal) and phalanges (PP: Proximal phalanx, IP: Intermediate phalanx) lengths (illustrated with mature males *P. troglodytes* ). Measurements are illustrated on the third manual and pedal rays. The protocol was identical for all other rays.

All measurements were taken on digital copies of the original radiographs, available on *Morphosource* (Thompson et al. 2020). Using the software *ImageJ* (version 1.54 g), we measured the proximal‐distal length of bones within the hand and foot with the “segmented line tool”. Hand length was measured from the most proximal point of the lunate to the tip of the third digit, that is, the tip of the distal phalanx, and foot length was measured from the most proximal point of the calcaneus to the tip of the third digit. We measured metapodials and phalanges from each ray, excluding the distal phalanges to be consistent with previous studies (Figure 1; see Lemelin 1999; Young and Heard‐Booth 2016; Druelle et al. 2017; Young et al. 2019; Toussaint et al. 2025). All measurements were in pixels. When a calibration scale was available on the radiographs (322 of 355 cases), we converted the measurements to centimeters and used them for the allometric analysis. All radiographs, regardless of scaling, were used for the phalangeal index calculation (see below). Due to the low quality of some radiographs, we did not attempt to measure rays that were obscured, in superposition, or from damaged radiographs.

From these data we calculated intrinsic digital proportions with the phalangeal index (PI) for each digit (i.e., PIs: summed length of non‐distal phalanges relative to corresponding metapodial length; e.g., Kirk et al. 2008; Lemelin 1999), as follows:

$$\mathrm{PI}=\frac{\text{proximal phalanx}+\text{intermediate phalanx}}{\text{metapodial}}\times 100$$

### 2.3 Statistics

#### 2.3.1 Investigating Allometry in Hands and Feet

To investigate the ontogenetic allometry of hand and foot lengths, we performed bivariate log–log regressions against total body mass using Reduced Major Axis (RMA) models, which allow residual variance in both plotted traits to be taken into account. This approach is commonly used in allometric studies (e.g., Lande 1979; Jungers and Fleagle 1980; Sokal and Rohlf 1995; White and Seymour 2005). However, because our dataset is built from longitudinal data, controlling for repeated measurements of the same individual can be relevant. We therefore also applied Linear Mixed Effects (LME) models, including sex as an independent variable and individual identity as a random intercept. As individual body mass data were not available, we used the mean body mass values reported by Gavan (1971) and provided by age and sex derived from the 15 chimpanzees used in our study. Regression slopes ±95% confidence intervals (CI) were compared with the isometric expectation of 0.333 for length (a linear measure) versus mass (a volumetric measure). Ontogenetic scaling was qualified as negative allometry, positive allometry, or isometry when the 95% CIs about the slope were greater than, less than, or inclusive of 0.333, respectively. Furthermore, we applied the same methodology for each bone from each ray of manual and pedal digits. Finally, we applied non‐linear regression modeling using a Gompertz model to test our prediction that ontogenetic changes in manual and pedal rays could be explained by distinct growth patterns of metapodials and phalanges. Each bone length was fit to a Gompertz model of growth, as follows:

$$y=Ae^{-be^{-\mathrm{kt}}}$$

where y is the bone length of interest, *t* is the age in days, *A* is the asymptotic size at maturation, and *b* and *k* are scaling parameters proportional to the initial growth rate and the rate of growth deceleration (see Farmer and German 2004; Fiorello and German 1997; German and Meyers 1989). From these fitted growth curves, we extracted key developmental indicators: the time to growth completion (*Tf*) and the maximum growth rate (*Rmax*), both derived from the first derivative of the Gompertz function, as follows:

$$\frac{\mathrm{dy}}{\mathrm{dt}}=A{\mathrm{bke}}^{-be^{-\mathrm{kt}}}e^{-\mathrm{kt}}$$

The peak growth rate (*Rmax*) was computed as the maximum of the first derivative of the Gompertz growth function, equivalent to the expression Ake^−2^ (Fiorello and German 1997). To define the end of the growth period (*Tf*), we identified the age at which the instantaneous growth rate dropped to 20% of its maximum value. This threshold was selected rather than the previously used 5% (e.g., *Cebus albifrons* : Young and Heard‐Booth 2016) to better reflect the extended developmental period typical of great apes like the chimpanzee, whose skeletal growth continues over a longer timeframe compared to small‐bodied primates. Additionally, this value was selected due to the lack of measurements from really early ages (< 1.5 years old).

#### 2.3.2 Evaluating Ontogenetic Changes in Intrinsic Digit Proportions

To test our prediction that intrinsic digit proportions decline during chimpanzee growth, we fit LME models between age*sex and phalangeal indices, fitting separate models for each manual and pedal digit. Both phalangeal index (dependent variable) and age (independent variable) were logarithmically transformed prior to analysis to improve bivariate normality and linearize the growth‐related relationship between proportional changes and age, following previous studies (e.g., Young and Heard‐Booth 2016). Individual identity was included as a random effect to account for repeated measures taken from the same individuals throughout ontogeny. This random structure corrects for the non‐independence of longitudinal data and provides more reliable estimates of population‐level trends.

All statistical analyses were performed using the *R* statistical platform (R Core Team 2025), supplemented by the add‐on packages *lme4* (Bates et al. 2015), *lmerTest* (Kuznetsova et al. 2015), *plyr* (Wickham and Wickham 2019), and *dplyr* (Wickham et al. 2014).

## 3 Results

### 3.1 Ontogeny of Hand and Foot Lengths in *Pan troglodytes*

**FIGURE 2:**
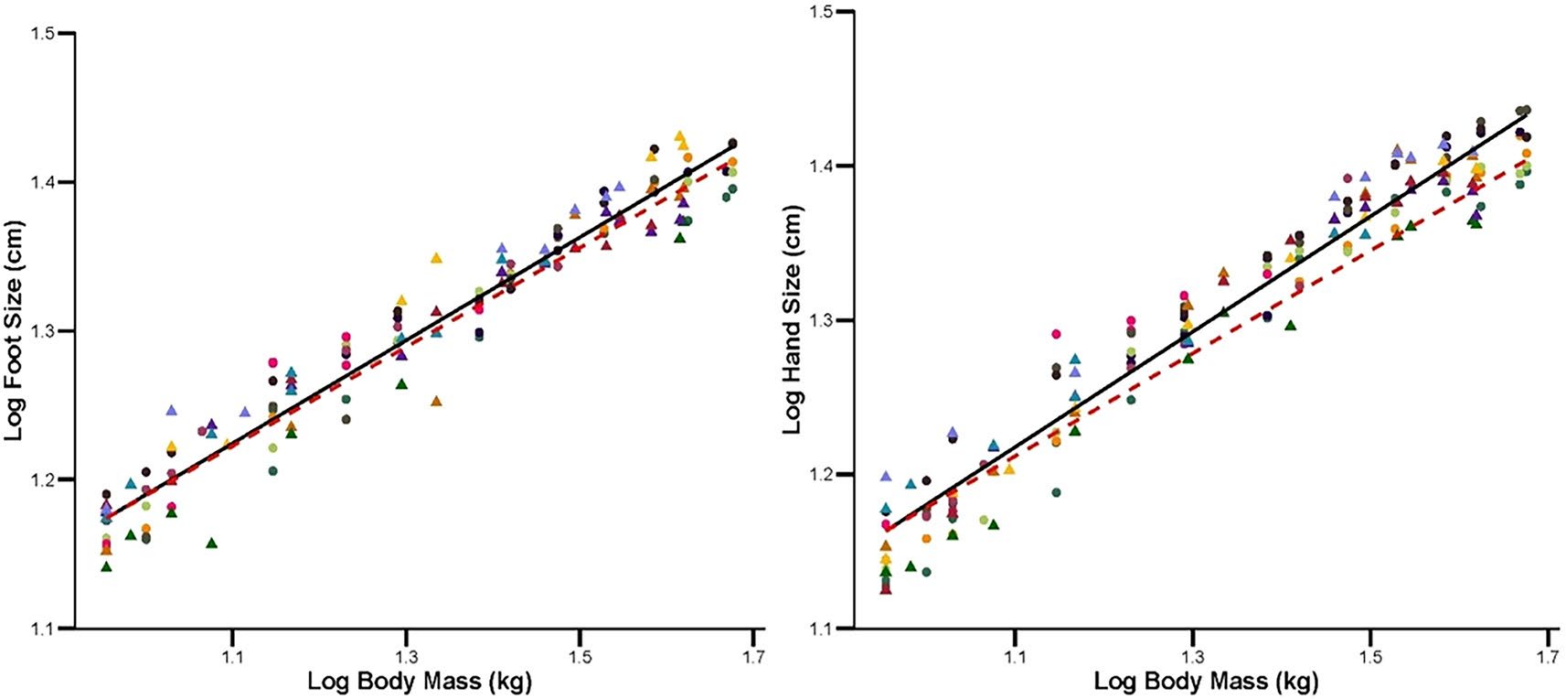
Allometric relationships between hand and foot lengths and body mass in growing male (circles) and female (triangles) *P. troglodytes*. The data are plotted on a log–log scale and the allometry is quantified using linear mixed‐effects (LME; black line). Note that the Reduced Major Axis regression models are not shown here because it is nearly identical. Various colors indicate individual chimpanzees. The red dashed line in each panel indicates isometric scaling (i.e., a scaling exponent of 0.333).

Chimpanzee hands and feet grow with positive allometry (Figure 2) and there is no sex effect (*p* = 0.51 and *p* = 0.31; respectively). The scaling exponents are 0.374 (LME: 0.363–0.384; RMA: 0.362–0.390) for the hand and 0.346 (LME: 0.334–0.357; RMA: 0.333–0.361) for the foot (Table 2). These values are consistent across both RMA and LME estimation methods, though 95% CIs were slightly different, with the foot being very close to isometric scaling. Adult chimpanzees exhibit relatively longer hands and feet than juveniles.

**TABLE 2:** Ontogenetic allometry of *P. troglodytes* hand and foot lengths using Linear Mixed‐Effects (LME) regression models and Reduced Major Axis (RMA) regression models.

	Hand	Foot
LME‐Scaling exponent	0.374	0.346
(95% CI)	(0.363, 0.384)	(0.334, 0.357)
*R* ^*2*^	0.97	0.96
Adjusted *p*‐value	< 0.001	< 0.001
RMA‐Scaling exponent	0.376	0.346
(95% CI)	(0.362, 0.390)	(0.333, 0.361)
*R* ^*2*^	0.95	0.95
Adjusted *p*‐value	< 0.001	< 0.001

### 3.2 Allometry of Metapodials and Phalanges During Ontogeny

**FIGURE 3:**
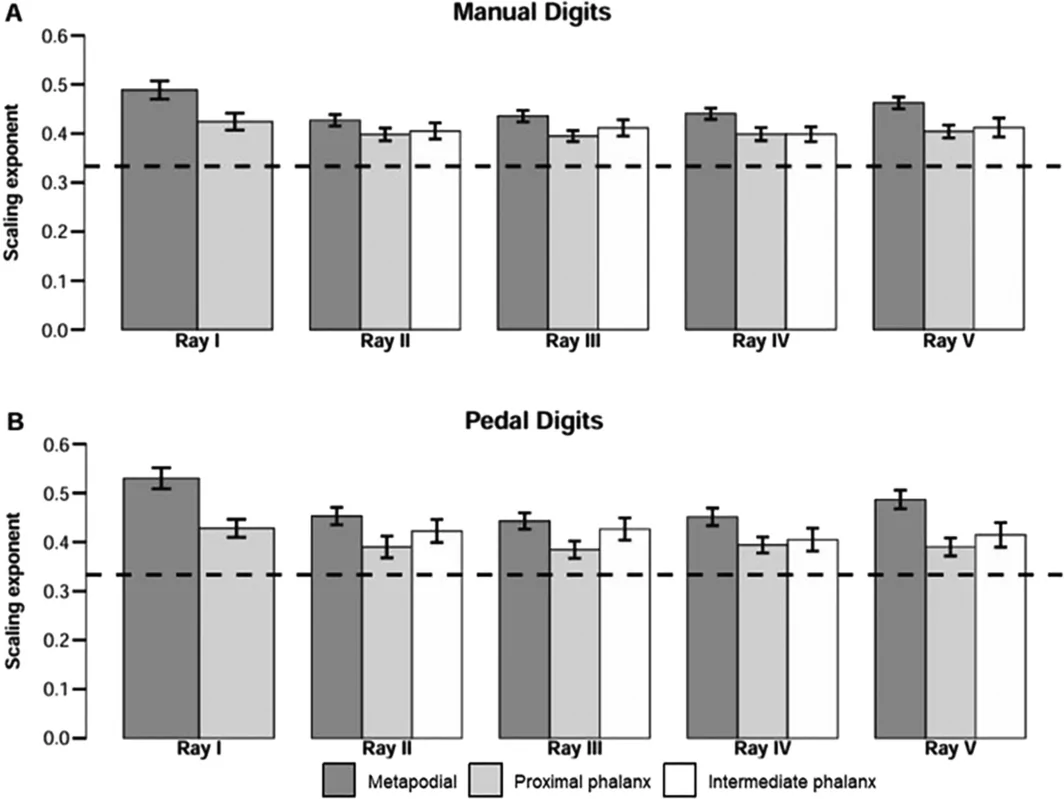
Ontogenetic allometry of metapodial and phalangeal lengths in the hand (A) and foot (B) relative to body mass in *P. troglodytes* . Bar heights indicate scaling exponents, including error bars representing 95% CI. The bold dashed horizontal line in each panel indicates the isometric scaling, that is, a scaling exponent of 0.333.

When examining allometric scaling exponents at the level of individual skeletal elements, all bones exhibited significant positive allometry (Table 3). The most pronounced positive allometry was observed in the metacarpals and metatarsals, generally followed by the intermediate phalanges, with the proximal phalanges showing the lowest scaling exponents (Figure 3; Table 3). Table 4 summarizes the sex‐related differences detected in the hand and foot for intermediate and proximal phalanges, as well as the metapodials. These differences reveal higher magnitude of positive allometry of males compared to females for these bones. However, the positive allometry was always detected in both females and males.

**TABLE 3:** Ontogenetic allometry of *P. troglodytes* digital rays (based on LME models).

		Digit 1		Digit 2		Digit 3		Digit 4		Digit 5	
		Manual	Pedal	Manual	Pedal	Manual	Pedal	Manual	Pedal	Manual	Pedal
Metapodials	Scaling exponent	0.489	0.53	0.427	0.453	0.435	0.443	0.44	0.452	0.462	0.487
	95% CI on scaling	0.47	0.509	0.415	0.435	0.424	0.426	0.429	0.434	0.45	0.468
	Exponent	0.507	0.552	0.439	0.471	0.447	0.46	0.452	0.469	0.474	0.506
	Scaling trend	+	+	+	+	+	+	+	+	+	+
	*R* ^*2*^	0.897	0.915	0.946	0.918	0.953	0.925	0.957	0.921	0.959	0.924
	Adjusted *p*‐value	< 0.001	< 0.001	< 0.001	< 0.001	< 0.001	< 0.001	< 0.001	< 0.001	< 0.001	< 0.001
Proximal phalanges	Scaling exponent	0.424	0.428	0.398	0.39	0.395	0.384	0.399	0.394	0.404	0.39
	95% CI on scaling	0.407	0.41	0.385	0.368	0.384	0.367	0.386	0.378	0.391	0.372
	Exponent	0.441	0.446	0.411	0.412	0.406	0.402	0.412	0.411	0.417	0.409
	Scaling trend	+	+	+	+	+	+	+	+	+	+
	*R* ^*2*^	0.85	0.892	0.922	0.854	0.949	0.894	0.944	0.896	0.938	0.842
	Adjusted *p*‐value	< 0.001	< 0.001	< 0.001	< 0.001	< 0.001	< 0.001	< 0.001	< 0.001	< 0.001	< 0.001
Intermediate phalanges	Scaling exponent	−	−	0.405	0.423	0.411	0.427	0.399	0.405	0.412	0.415
	95% CI on scaling	−	−	0.389	0.399	0.395	0.404	0.383	0.382	0.393	0.39
	Exponent	−	−	0.422	0.446	0.428	0.449	0.414	0.428	0.432	0.44
	Scaling trend	−	−	+	+	+	+	+	+	+	+
	*R* ^*2*^	−	−	0.88	0.834	0.882	0.832	0.892	0.826	0.879	0.793
	Adjusted *p*‐value	−	−	< 0.001	< 0.001	< 0.001	< 0.001	< 0.001	< 0.001	< 0.001	< 0.001

**TABLE 4:** Sex‐related differences in the allometric scaling exponent of metapodials and phalanges of the hand and foot during ontogeny.

	Hand			Foot		
	Slope* female	Slope male	Female versus male: adjusted *p* value	Slope female	Slope male	Female versus male: adjusted *p value*
M 1	0.47	0.50	0.2401	0.51	0.55	**0.0498**
PP 1	0.41	0.43	0.5988	0.42	0.43	0.2949
M 2	0.43	0.43	0.9761	0.42	0.48	**0.0091**
PP 2	0.39	0.41	0.1613	0.36	0.41	**0.0479**
IP 2	0.38	0.43	**0.0341**	0.43	0.42	0.8659
M 3	0.44	0.44	0.8237	0.42	0.46	**0.0492**
PP 3	0.39	0.40	0.7476	0.36	0.40	**0.0498**
IP 3	0.39	0.43	**0.0341**	0.40	0.45	**0.0230**
M 4	0.44	0.44	0.9761	0.43	0.47	**0.0492**
PP 4	0.39	0.40	0.4119	0.38	0.40	0.1543
IP 4	0.37	0.42	**0.0341**	0.37	0.44	**0.0091**
M 5	0.45	0.47	0.1301	0.46	0.50	**0.0479**
PP 5	0.39	0.41	0.1148	0.39	0.39	0.6765
IP 5	0.38	0.44	**0.0341**	0.42	0.41	0.7109

* The slope corresponds to the allometric scaling exponent based on LME models. M: metapodials, PP: Proximal Phalanx, IP: Intermediate Phalanx. Significant results are indicated in bold and highlighted in gray.

A total of 28 Gompertz models were fitted to the hand and foot growth data (see Table B in Supporting Information), each corresponding to one bone segment (metapodial, proximal and intermediate phalanges) from rays I to V of the hand and foot. When comparing average growth cessation time (*Tf*) across bone segments, metapodials exhibited the longest growth durations (mean *Tf* = 10.5 ± 0.48 years), followed by proximal phalanges (10.41 ± 0.70 years) and intermediate phalanges (9.72 ± 0.48 years). Peak growth velocity (*Rmax*) presents a very similar pattern across bone segments: intermediate phalanges displayed the highest average values (0.026 ± 0.0012 cm/day), followed closely by metapodials (0.025 ± 0.0012 cm/day) and proximal phalanges (0.024 ± 0.0018 cm/day).

Comparison between manual and pedal elements showed differences between hands and feet. Growth duration (*Tf*) was longer in proximal phalanges of the hand compared to the foot (mean *Tf* = 10.85 ± 0.42 years and 9.96 ± 0.81 years, respectively). Similarly, manual metacarpals grow for a longer time than pedal metatarsals (mean *Tf* = 10.63 ± 0.46 years and 10.37 ± 0.47 years, respectively). For intermediate phalanges, the pedal elements had slightly higher *Tf* values than the manual ones (10.08 ± 0.66 vs. 9.36 ± 0.16 years, respectively). *Rmax* values of the metapodials are relatively similar between hands and feet (0.025 and 0.026 cm/day, respectively). Phalanges exhibit different patterns: in the foot, proximal and intermediate phalanges have similar peak growth velocities (0.025 cm/day), whereas in the hand, proximal phalanges have a lower peak growth velocity than intermediate phalanges (0.023 and 0.027 cm/day, respectively).

### 3.3 Changes in Phalangeal Index During Growth

**FIGURE 4:**
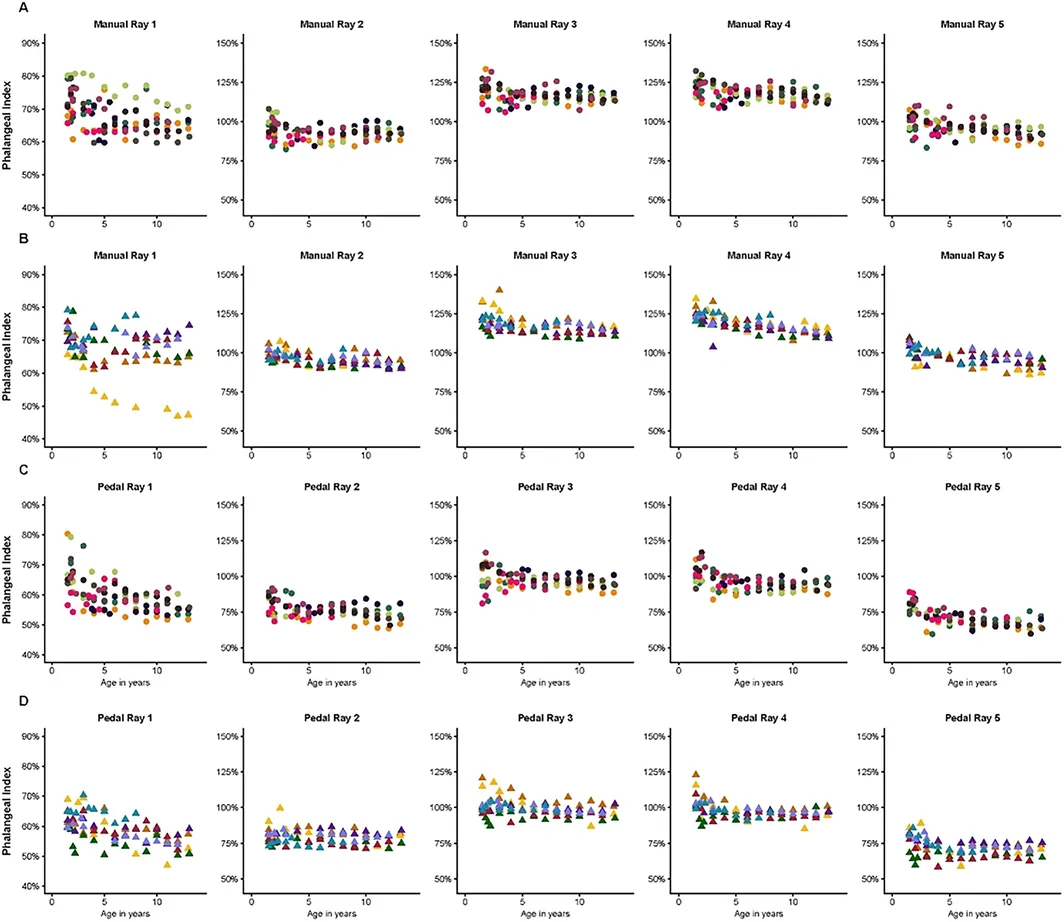
Ontogenetic changes in manual and pedal phalangeal indices in female (triangles) and male (circles) *P. troglodytes*. Various colors indicate individual chimpanzees.

Manual and pedal phalangeal indices decline significantly throughout postnatal growth in both male and female *P. troglodytes* , indicating that chimpanzees possess relatively more elongated digits compared to metapodials at an early age (Table 5; Figure 4). While the general pattern of developmental change is consistent across individuals, interindividual variation is evident and particularly notable in one female for manual ray 1, which exhibits a substantial decrease in the phalangeal index. The only significant sex difference is observed in the phalangeal index of digit 2, which remains significantly higher in females (i.e., relatively longer phalanges) than in males during ontogeny (*p =* 0.0026). During the growth period, phalangeal indices in the pollex and hallux declined by 8% and 9%, respectively. Across digits II–V, manual phalangeal indices decreased by an average of 9%, while pedal indices decreased by an average of 7% (Table 5).

**TABLE 5:** Ontogenetic changes in *P. troglodytes* (males and females together) phalangeal indices.

	Ray 1		Ray 2		Ray 3		Ray 4		Ray 5	
	Manual	Pedal	Manual	Pedal	Manual	Pedal	Manual	Pedal	Manual	Pedal
Slope	−0.536	−0.842	−0.350	−0.545	−0.557	−0.583	−0.730	−0.639	−0.752	−0.786
*R* ^*2*^	0.599	0.508	0.270	0.533	0.362	0.473	0.411	0.352	0.455	0.431
Adjusted *p*‐value	< 0.001	< 0.001	< 0.001	< 0.001	< 0.001	< 0.001	< 0.001	< 0.001	< 0.001	< 0.001
PI at 1.5 years	73%	64%	98%	81%	121%	102%	124%	105%	103%	79%
(95% CI)	(70%, 76%)	(61%, 68%)	(95%, 102%)	(78%, 85%)	(117%, 125%)	(96%, 108%)	(120%, 127%)	(99%, 110%)	(100%, 106%)	(76%, 83%)
PI at 13 years	65%	55%	92%	77%	115%	96%	113%	96%	92%	69%
(95% CI)	(59%, 70%)	(52%, 57%)	(90%, 94%)	(71%, 82%)	(113%, 117%)	(92%, 100%)	(111%, 115%)	(92%, 99%)	(89%, 94%)	(66%, 73%)
Percent Change	−11%	−15%	−6%	−6%	−5%	−5%	−9%	−9%	−11%	−13%

## 4 Discussion

Contrary to previous findings on primates (e.g., Druelle et al. 2017; Jungers and Fleagle 1980; Lawler 2006; Young and Heard‐Booth 2016), rodents (Young et al. 2019), and mammals in general (Young et al. 2019), chimpanzees exhibit a positively allometric relationship between both hand and foot lengths with body mass during ontogeny. As a result, adult male and female chimpanzees have relatively longer hands and feet for their body size compared to younger chimpanzees. At first glance, this may seem to contradict earlier results on chimpanzee growth, where negative allometry of hand and foot growth was observed (e.g., Schoonaert et al. 2007; Schultz 1940). However, the method used for scaling is an important factor here. Indeed, whether body mass (as in this study) or stature (or trunk) length is taken as the reference variable (see Schoonaert et al. 2007; Schultz 1940) can lead to different interpretations. When hand length is considered relative to stature length, the same reduction in relative length reported by Schoonaert et al. (2007) and Schultz (1940) is observed in the present study. In contrast, when body mass is used to estimate allometry, as it is commonly done in ontogenetic studies, a slight but significant positive allometry is observed. Jungers and Fleagle (1980) already pointed to the influence of the scaling factor used by emphasizing that trunk or stature length is very likely to not scale isometrically with body mass during growth.

The positive allometry of hands and feet relative to body mass aligns with the ontogenetic scaling patterns of metapodials and phalanges. Our results show that all metapodials, as well as both proximal and intermediate phalanges of the hand and foot, exhibit positive allometry during ontogeny. However, the magnitude of this allometry varies among skeletal elements and between sexes. Metapodials display the strongest positive allometry, followed by intermediate and then proximal phalanges. Consequently, the proportional increase in metapodial length outpaces that of phalangeal length, leading to a progressive decline in both manual and pedal phalangeal indices throughout postnatal growth. This pattern is consistent with findings in other primates (Young and Heard‐Booth 2016; Druelle et al. 2017). While the overall positive allometry of chimpanzee autopodia appears unique, the differential scaling pattern among elements mirrors that of other primates, with similar specific differences in digit scaling (Young and Heard‐Booth 2016; Druelle et al. 2017). Although sex‐related differences exist in the magnitude of the allometry of specific metapodials and phalanges, it does not change the nature of the allometry (the core scaling relationship is preserved) and the resulting phalangeal indices remain remarkably similar between males and females (except for digit II in the hand; see Nelson and Shultz 2010 for a possible explanation of this difference). As previously shown (Druelle et al. 2017; Huxley 1942; Turnquist and Kessler 1989) and initially hypothesized by Huxley (1942), differences in body dimensions during growth are the result of overall body size variation (e.g., sexual dimorphism) rather than proportional changes between the sexes.

To complement these elements, nonlinear modeling of bone growth showed differences in growth dynamics among autopodial elements. In the hand, proximal phalanges displayed the longest growth duration (*Tf*) but the lowest peak growth rates (*Rmax*), followed by the metacarpals, whereas intermediate phalanges exhibited shorter growth periods but higher growth velocity peaks. In the foot, metatarsals display the longest growth duration and the highest peak growth rates compared to proximal and intermediate phalanges. These results indicate that both growth timing and rate contribute to the ontogenetic shifts observed in digit proportions. One might wonder whether these observations are related to the sequence of epiphyseal fusion. To explore this, a closer examination of the fusion period would be needed. However, based on the present radiographs, it appears that the intermediate phalanges generally fuse before the metapodials. Comparisons between manual and pedal elements further highlight interlimb differences. Manual bones generally show longer growth durations than their pedal counterparts, suggesting a more extended developmental trajectory for the hand relative to the foot. However, this pattern reverses for the intermediate phalanges, where pedal elements continue growing slightly longer than manual ones. Despite these differences in timing, peak growth rates remain broadly similar between hands and feet (from 0.023 to 0.027 cm/day). Together, these findings suggest that the hand continues to grow for a longer time than the foot, potentially explaining the stronger positive allometry observed in the hand. Also, as previously documented in *Cebus albifrons* , *Sapajus apella* , and *Papio anubis*, manual rays are generally characterized by higher phalangeal indices than pedal rays (Young and Heard‐Booth 2016; Druelle et al. 2017).

### 4.1 Reflecting Functional Adaptations Through Development

The specific ontogenetic pattern now described for chimpanzee hands and feet likely reflects selective pressures related to the interaction between their large body size and their ecological niche, including their locomotor repertoire. Among specialized grasping animals, large and long hands and feet have been hypothesized to enhance grasping performance by increasing the contact surface of autopodia (e.g., Grand 1977; Jungers and Fleagle 1980; Lawler 2006; Preuschoft 2004). For large species, foraging in the trees poses a challenge, and relatively long grasping extremities should enhance arboreal performance. Interestingly, the intensity of allometry is stronger in the hand than in the foot, which scales almost isometrically. Hence, juvenile feet are proportionally more similar to adult feet than juvenile hands are to adult hands, suggesting distinct selective pressures acting on the hands and feet (e.g., Patel et al. 2015; Druelle et al. 2018). The greater relative increase in hand length is likely to compensate the substantial increase in body mass, allowing effective grasping, climbing and suspensory performance in adulthood. Furthermore, the main changes in phalangeal indices appear to stabilize around the age of five years across both manual and pedal digits (see Figure 4). This age corresponds to the developmental stage when young chimpanzees begin to exhibit an adult‐like locomotor repertoire, characterized by increased knuckle‐walking and a significant decline in torso‐orthograde suspensory behaviors (Doran 1992; Sarringhaus et al. 2014). Figure 4 also reveals that variability in phalangeal indices is greater during infancy and progressively converges with age, suggesting a canalization process toward a more restrained adult morphology. Canalization is often associated with stabilizing selection, where reduced variability reflects strong functional constraints on a given trait. Indeed, strong and consistent pressures should tend to canalize morphological variation to ensure functional reliability, while more heterogeneous functional demands should tend to relax stabilizing selection (e.g., Donihue et al. 2018). In the case of chimpanzees, the decreasing variance observed with age likely reflects stabilizing selection on digit proportions. As juveniles grow, functional trade‐offs emerge between maintaining efficient arboreal locomotion for foraging and enabling terrestrial travel over longer distances (Pontzer and Wrangham 2004). Given their relatively large body mass, both male and female adult chimpanzees cannot afford substantial deviations in phalangeal proportions without compromising both grasping and climbing efficiency on the one hand, and knuckle‐walking performance on the other hand. Thus, the canalization observed in our ontogenetic data is likely to represent the outcome of strong stabilizing selection, acting to maintain uniformity in autopodial morphology among adult chimpanzees.

Furthermore, chimpanzees (like orangutans) exhibit hands with particularly elongated digits compared to other apes (Almécija et al. 2015). Given the calculated phalangeal indices (85% for digits II‐V in the foot and 103% for digits II‐V in the hand), the metacarpals appear particularly elongated. This likely reflects a compromise between arboreal and terrestrial locomotion, with knuckle‐walking in the hands and plantigrady in the feet. A recent longitudinal study of hand bone allometry in humans showed a similar pattern of positive allometry, suggesting a shared developmental pattern among hominids (Kondo et al. 2017). However, in humans, the strongest positive allometry is observed in the intermediate phalanges, followed by the metacarpals and proximal phalanges of digits II–V. This pattern is reversed in digit I, where the proximal phalanx exhibits stronger positive allometry than the metacarpal, a difference that may be related to the unique manipulative abilities of the human hand, including the important functional role of the thumb (e.g., Almécija et al. 2015; Karakostis et al. 2021; Bardo et al. 2020; Bardo et al. 2024).

The variability in intrinsic proportions among primates (see Almécija et al. 2015; Toussaint et al. 2025) suggests that adjustments in the growth patterns of hand and foot proportions may represent a process on which selection can act, allowing for the development of adult morphologies that respond to various functional demands. This study on chimpanzees thus provides a framework for speculating on the evolutionary processes shaping hand and foot proportions. Interestingly, Miocene apes such as *Hispanopithecus laietanus* exhibit higher phalangeal indices than chimpanzees, indicating relatively shorter metapodials (Almécija et al. 2007). This pattern may result from metapodial isometry, potentially facilitated by reduced limb integration, which is a trait described in hominoids (Young et al. 2010). Such reduced integration may also extend to the hands and feet, contributing to the important variability observed among extant hominoids (Almécija et al. 2015).

### 4.2 Chimpanzees Versus Capuchin Monkeys and Baboons

**FIGURE 5:**
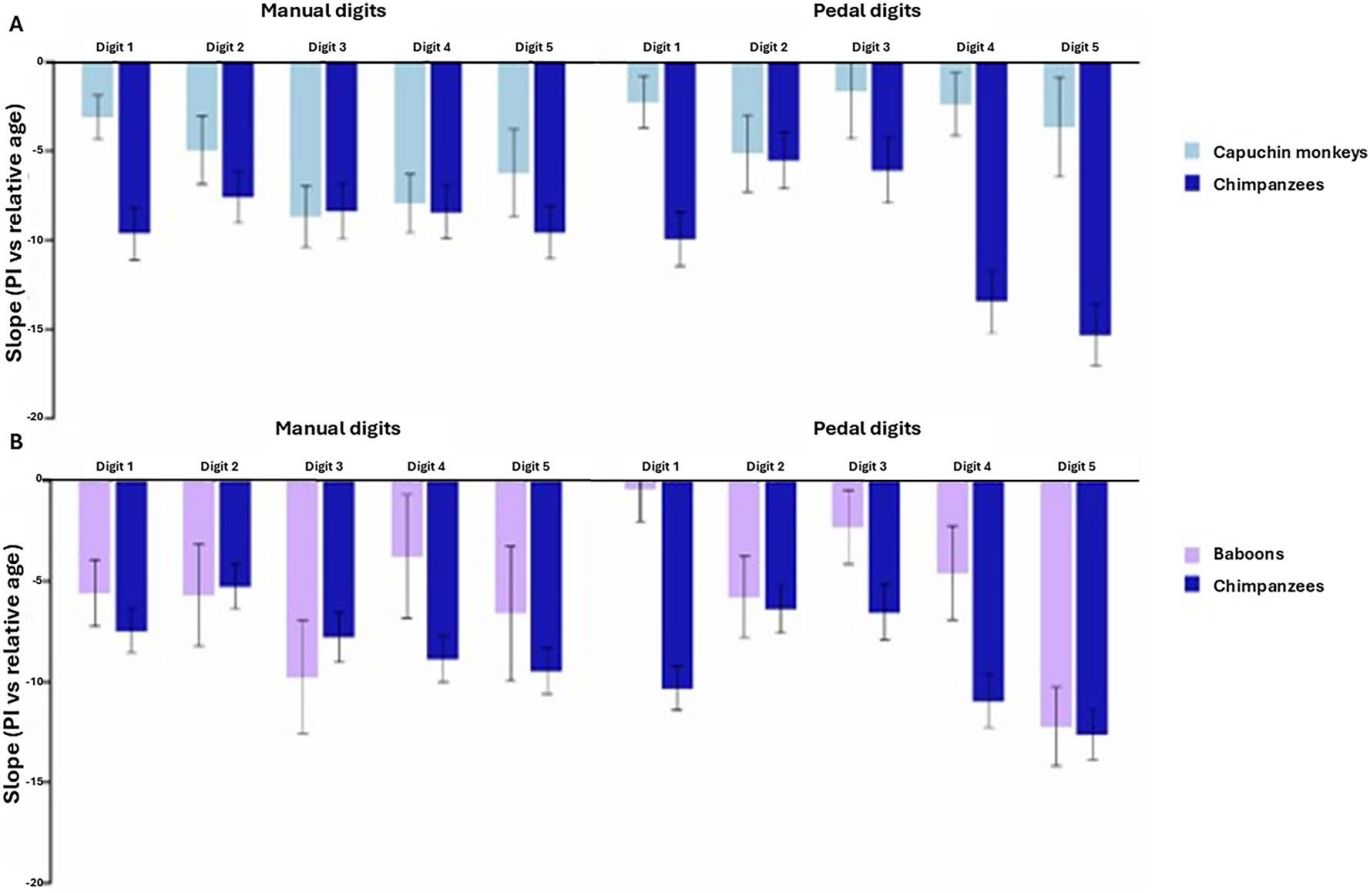
Estimated slopes of linear mixed‐effects regressions of phalangeal indices vs. relative age based on the end of growth. (A) For *Cebus albifrons*, the end of growth is estimated at 6.5 years, with data available from 31 days (0.08 years) to 1574 days (4.3 years; Young and Heard‐Booth 2016), corresponding to 1% to 66% of growth. The present chimpanzee data cover 11.5% (1.5 years) to 100% (13 years) of growth. Comparisons between species are thus based on the overlapping period of 11.5% to 66% of growth. (B) For *Papio anubis*, the end of growth is estimated at 7 years, with data available from 0.42 years (6%) to 6.4 years (91%; Druelle et al. 2018; Toussaint et al. 2025). Comparisons with chimpanzees were thus possible for the overlapping period of 11.5% to 91% of growth. Error bars indicate 95% confidence intervals on the estimate.

Currently, comparable developmental data on digit growth are available for baboons, which are large and terrestrial, and capuchin monkeys, which are small and predominantly arboreal. Given the importance of climbing and suspensory locomotion in adult chimpanzees and their large body size, we expected that they would exhibit a slower decline in intrinsic autopodial proportions during growth than baboons and capuchins, thereby retaining more juvenile‐like grasping morphologies into adulthood. However, Figure 5 shows this is not the case. The magnitude of reduction in phalangeal index is comparable between chimpanzees and capuchins for all manual digits (based on standard deviation overlap), except for the pollex, which declines more sharply in chimpanzees. For pedal digits, chimpanzees exhibit a greater reduction in rays I, IV and V. When comparing chimpanzees to baboons, the magnitude of reduction in the phalangeal index is comparable for all manual digits, except for digit IV, where chimpanzees show a steeper decline. In the foot, chimpanzees exhibit a greater overall reduction in rays I, III and IV.

Note that although we attempted to normalize the developmental periods to allow comparisons, these trends must be interpreted cautiously due to substantial differences in life history traits, particularly growth rate and longevity. Nevertheless, chimpanzees, which are large primates that bear most of their weight on their hindlimbs (Raichlen et al. 2009) and spend a significant proportion of their daily activities walking on the ground (Pontzer and Wrangham 2004), should experience substantial mechanical pressures on distal limb segments during growth. This would result in stronger reductions in foot proportions compared to baboons and capuchin monkeys. Here, this reduction is related to the development of long (and robust) metatarsals.

Additionally, because muscle force and bone rigidity scale with cross‐sectional area (∝ length^2^), while body mass scales with volume (∝ length^3^), small arboreal primates, such as capuchin monkeys (< 4 kg), should exhibit higher mass‐specific power output and greater bone robusticity. This would reduce mechanical pressures on the autopodia during development (Toussaint et al. 2025). Consequently, capuchin monkeys may face less selective pressure than chimpanzees, and thus the former may retain juvenile‐like grasping morphologies into adulthood, as reflected in the lower slopes observed in foot proportions (Figure 5). Baboons appear to be intermediate between capuchins and chimpanzees in this regard.

With regard to the hands, the trends are more similar across species and cannot be explained by locomotor constraints alone. Interestingly, chimpanzees, capuchins, and baboons share a broadly similar pattern of manual phalangeal reduction. This convergence suggests common selective pressures on the hand, likely reflecting a compromise between maintaining arboreal grasping ability across different locomotor modes and preserving manipulative capabilities.

Overall, primates exhibit distinct patterns of phalangeal index reduction in the foot. Although all species examined show declines in relative phalangeal length during postnatal growth, the magnitude, timing, and anatomical distribution of these changes vary. These differences, which also appear to occur within a broader mammalian pattern (Young et al. 2019), likely reflect adaptive ontogenetic trajectories shaped by each species' ecological demands and locomotor constraints. The modulation of autopodial proportions during development may thus represent a key evolutionary mechanism in primate diversification and helps in the functional interpretation of fossil taxa. Future research incorporating broader taxonomic sampling, longitudinal growth data, and integrative behavioral analyses will be crucial to further clarify the evolutionary pathways of primate autopodial morphology.

## Author Contributions

**Tom Combemorel:** writing – original draft, methodology, validation, visualization, software, formal analysis, data curation, investigation, resources. **Jesse W. Young:** methodology, validation, writing – review and editing, software, investigation. **François Druelle:** conceptualization, investigation, writing – review and editing, resources, supervision, project administration, data curation, methodology, validation.

## Funding

The authors have nothing to report.

## Conflicts of Interest

The authors declare no conflicts of interest.

## Supporting information


**Table A**. Sample used in this study.
**Table B**. Gompertz parameters describing *P. troglodytes* digital ray growth.
**Figure A**. Allometric relationships between hand and foot lengths and body mass in growing male and female *P. troglodytes* per individual (*n* = 15). Data are plotted on a log–log scale and the allometry for the full group is quantified using A: linear mixed‐effects (LME; black line) and B: Reduced Major Axis (RMA; blue line) regression models. The red dashed line in each panel indicates isometric scaling (i.e., a scaling exponent of 0.333).

## Data Availability

All the data analyzed in this study are freely available on *MorphoSource* (www.morphosource.org). To access them, navigate to the “Teams & Projects” tab and search for “*The Nissen‐Riesen Chimpanzee Radiological Growth Series*” (see Thompson et al. 2020), which contains 3576 media files. The radiographs used in this study can be identified by entering the specimen numbers listed in Table A (Supporting Information). While additional files may appear in the search results, only hand and foot radiographs were analyzed in this paper.
