# Supplementary material for: Ontogenetic Changes in Intrinsic Hand and Foot Proportions in Chimpanzees, Pan troglodytes
Source: Am J Biol Anthropol. 2026 Sep 3;191(1):e70346. doi: 10.1002/ajpa.70346 (PMC13542625; doi:10.1002/ajpa.70346)
Supplement: Supplementary file 1 [file AJPA-191-e70346-s001.docx]

| **Individual** | **Sex** | **Age (in yrs) for which foot radiographs were captured** | **ImageIDFoot (File name in Morphosource)** |
| --- | --- | --- | --- |
| Alf | Male | 3.8; 4; 5; 5.5; 6; 7; 8; 9; 10; 11; 13 | 709; 850; 477; 699; 855; 183; 541; 855; 198; 474; 13 |
| Art | Male | 1.5; 1.8; 2; 3; 4; 5; 6; 7; 8; 9; 10; 11; 12; 13 | 151; 256; 383; 948; 716; 358; 797; 61; 475; 804; 153; 438; 696; 989 |
| Banka | Female | 1.5; 1.8; 2; 2.2; 3; 4; 5; 6; 7; 9; 10; 12; 13.1 | 914; 99; 285; 490; 6; 534; 898; 267; 590; 224; 26; 273 |
| Bard | Male | 1.5; 1.8; 2; 2.2; 3; 3.8; 5; 6; 7; 8; 9; 10; 11; 12 | 427; 541; 685; 830; 406; 920; 633; 946; 337; 647; 997; 262; 571; 831 |
| Falla | Female | 1.5; 2; 2.8; 3; 6; 7; 8; 9; 10; 11; 12 | 872; N-249; 609; 709; 719; 83; 346; N-651; 894; 113; 324 |
| Fanny | Female | 1.5; 2; 3; 4; 5; 6; 7; 8; 9; 10; 11; 12 | N-390; N-771; 383; 804; 99; 502; 813; 166; 455; 707; 2; 212 |
| Flora | Female | 1.5; 2.2; 3; 4; 6; 7; 8; 9; 10; 11; 12; 13.1 | 982; 562; N-51; N-57; N-60; N-65; 966; 246; 554; 809; 47; N-81 |
| Jed | Male | 1.5; 1.8; 3; 4; 5; 7; 8; 9; 10; 11; 12; 13.1 | 129; 303; 203; 689; 998; 696; 54; 324; 623; 870; 91; 296 |
| Jenny | Female | 1.5; 2.2; 3; 4; 5; 7; 8; 9; 10; 11; 12; 13 | N-433; 856; 428; 143; 638; 350; 658; 6; 282; 584; 846; 69 |
| Jent | Male | 1.5; 1.8; 2; 3; 5; 6; 7; 8; 9; 10; 11; 12.1 | 832; 15; 188; 678; 381; 689; 48; 317; 608; 857; 80; 311 |
| Jojo | Female | 1.5; 2; 2.5; 3; 4; 5; 6; 8; 11; 12; 13 | 510; 778; 154; 549; 228; 717; 10; 713; 638; 882; 102 |
| Karla | Female | 1.5; 1.8; 2.3; 3; 3.5; 4; 5; 6; 7; 8 | 937; N-151; N-15; 752; 884; 26; N-19; 757; 110; N-25 |
| Ken | Male | 1.5; 1.8; 2; 3; 3.5; 4; 5; 6; 7; 8; 9; 10; 11; 12; 13 | 139; N-236; N-362; 924; 298; 684; 338; 786; 53; 451; 790; 140; 426; 682; 976 |
| Scarf | Male | 1.5; 1.8; 2; 3.3; 3.5; 4; 4.5; 5; 6; 7; 10 | 848; 42; 227; 134; 277; 502; 759; 876; 235; N-573; 507 |
| Web | Male | 1.8; 2; 2.3; 4.5; 5; 7; 8; 10; 11.1 | 456; 560; 696; 468; 611; 232; 538; N-35; 241 |

**Supplementary Material**

**Ontogenetic changes in intrinsic hand and foot proportions in chimpanzees, *Pan troglodytes***

Tom Combemorel, Jesse W. Young, François Druelle

**Table A.** Sample used in this study.

| **Individual** | **Sex** | **Age (in yrs) for which hand radiographs were captured** | **ImageIDHand (File name in Morphosource)** |
| --- | --- | --- | --- |
| Alf | Male | 3.8; 4; 4.5; 5; 5.5; 6; 7; 8; 9; 10; 11; 13 | 708; 851; 221; 476; 698; 854; 182; N-543; 853; 200; 475; 15 |
| Art | Male | 1.5; 1.8; 2; 3; 4; 5; 6; 7; 8; 9; 10; 11; 12; 13 | 150; 257; 382; 949; 715; 359; 795; 65; 476; 802; 155; 439; 698; 992 |
| Banka | Female | 1.5; 1.8; 2; 2.2; 3; 4; 5; 6; 7; 9; 10; 12; 13.1 | 916; 100; 286; 491; 5; 535; 900; 266; 589; 226; 27; 275 |
| Bard | Male | 1.5; 1.8; 2.2; 3; 3.8; 5; 6; 7; 8; 9; 10; 11; 12; 13 | 426; 542; 831; 405; 921; 632; 948; 336; 649; 999; 264; 573; 830; 61 |
| Falla | Female | 1.5; 2; 2.8; 3; 7; 8; 9; 10; 11; 12 | 873; N-250; 610; 710; 76; 348; 654; 892; 116; 327 |
| Fanny | Female | 1.5; 2; 3; 4; 5; 6; 7; 8; 9; 10; 11; 12 | N-391; N-770; 389; 805; 98; 501; 811; 167; 456; 708; 4; 214 |
| Flora | Female | 1.5; 2.2; 3; 4; 6; 7; 8; 9; 10; 11; 12; 13.1 | 981; N-561; 74; N-56; N-59; N-64; 964; 248; 556; 811; 50; 284 |
| Jed | Male | 1.5; 1.8; 2; 3; 4; 5; 7; 8; 9; 10; 11; 12; 13.1 | 128; 302; 513; 204; 689; 998; 700; 59; 326; 629; N-876; 94; 299 |
| Jenny | Female | 1.5; 2.2; 3; 4; 5; 7; 8; 9; 10; 11; 12; 13 | N-434; 855; 427; 144; 638; 348; 657; 8; 284; 586; 840; 72 |
| Jent | Male | 1.5; 1.8; 2; 3; 5; 6; 7; 8; 9; 10; 11; 12.1 | 833; 16; 189; 677; 380; 687; 46; 319; 611; 856; 83; 310 |
| Jojo | Female | 1.5; 2; 2.5; 3; 4; 5; 6; 8; 11; 12; 13 | 506; 777; 155; 550; 229; 718; 9; 707; 641; 880; 105 |
| Karla | Female | 1.5; 1.8; 2.3; 3; 3.5; 4; 6; 7; 8 | 936; 152; N-16; 753; 883; 36; 758; 112; N-26 |
| Ken | Male | 1.5; 1.8; 2; 3; 3.5; 4; 5; 6; 7; 8; 9; 10; 11; 12; 13 | 143; N-238; N-364; 925; 299; 685; 333; 783; 52; 447; 789; 142; 427; 684; 978 |
| Scarf | Male | 1.5; 1.8; 2; 3.3; 3.5; 4; 4.5; 5; 6; 7; 10 | 847; 37; 226; 135; 278; 503; 760; 878; 236; N-572; 510 |
| Web | Male | 1.8; 2; 2.3; 4.5; 5; 7; 8; 10; 11.1 | 457; 561; 697; 467; 610; 234; 543; N-38; 243 |

**Table B.** Gompertz parameters describing *P. troglodytes* digital ray growth.

|  |  | **Digit 1** |  | **Digit 2** |  | **Digit 3** |  | **Digit 4** |  | **Digit 5** |  |
| --- | --- | --- | --- | --- | --- | --- | --- | --- | --- | --- | --- |
|  |  | **Manual** | **Pedal** | **Manual** | **Pedal** | **Manual** | **Pedal** | **Manual** | **Pedal** | **Manual** | **Pedal** |
| **Metapodials** | *A* | 4.41* | 6.16 | 9.33 | 7.99 | 9.43 | 7.49 | 8.82 | 7.10 | 8.16 | 7.41 |
|  |  | 4.16 | 5.85 | 9.03 | 7.64 | 9.14 | 7.19 | 8.51 | 6.85 | 7.84 | 7.17 |
|  |  | 4.67 | 6.48 | 9.63 | 8.34 | 9.73 | 7.78 | 9.12 | 7.34 | 8.47 | 7.66 |
|  | *b* | 1.14 | 1.27 | 1.03 | 1.07 | 1.04 | 1.04 | 1.04 | 1.10 | 1.08 | 1.18 |
|  |  | 1.04 | 1.16 | 0.98 | 0.99 | 0.99 | 0.97 | 0.99 | 1.02 | 1.02 | 1.08 |
|  |  | 1.24 | 1.37 | 1.07 | 1.15 | 1.09 | 1.11 | 1.09 | 1.18 | 1.15 | 1.29 |
|  | *k* | 0.0007 | 0.0007 | 0.0007 | 0.0007 | 0.0007 | 0.0006 | 0.0006 | 0.0008 | 0.0006 | 0.0007 |
|  |  | 0.0005 | 0.0006 | 0.0006 | 0.0006 | 0.0006 | 0.0005 | 0.0006 | 0.0006 | 0.0006 | 0.0006 |
|  |  | 0.0008 | 0.0008 | 0.0008 | 0.0008 | 0.0008 | 0.0007 | 0.0007 | 0.0009 | 0.0007 | 0.0008 |
|  | *Tf* | 4048 | 3968 | 3632 | 3872 | 3695 | 3964 | 3994 | 3495 | 4025 | 3632 |
|  | *Rmax* | 0.024 | 0.026 | 0.026 | 0.025 | 0.026 | 0.024 | 0.024 | 0.028 | 0.024 | 0.027 |
|  | *R²* | 0.94 | 0.93 | 0.97 | 0.94 | 0.97 | 0.94 | 0.97 | 0.94 | 0.97 | 0.95 |
| **Proximal phalanges** | *A* | 2.94 | 3.32 | 5.48 | 3.86 | 6.41 | 4.39 | 5.96 | 4.29 | 4.70 | 3.38 |
|  |  | 2.73 | 3.19 | 5.22 | 3.71 | 6.17 | 4.23 | 5.75 | 4.10 | 4.51 | 3.21 |
|  |  | 3.15 | 3.45 | 5.75 | 4.01 | 6.65 | 4.56 | 6.17 | 4.48 | 4.88 | 3.55 |
|  | *b* | 0.98 | 1.05 | 0.93 | 0.94 | 0.93 | 0.92 | 0.94 | 0.92 | 0.94 | 0.90 |
|  |  | 0.91 | 0.97 | 0.87 | 0.84 | 0.88 | 0.85 | 0.88 | 0.86 | 0.88 | 0.83 |
|  |  | 1.05 | 1.12 | 0.99 | 1.04 | 0.98 | 0.99 | 1.00 | 0.99 | 1.00 | 0.97 |
|  | *k* | 0.0006 | 0.0008 | 0.0006 | 0.0008 | 0.0007 | 0.0007 | 0.0007 | 0.0006 | 0.0006 | 0.0006 |
|  |  | 0.0004 | 0.0007 | 0.0005 | 0.0006 | 0.0006 | 0.0006 | 0.0006 | 0.0005 | 0.0005 | 0.0005 |
|  |  | 0.0007 | 0.0009 | 0.0007 | 0.0009 | 0.0007 | 0.0008 | 0.0007 | 0.0007 | 0.0007 | 0.0007 |
|  | *Tf* | 4347 | 3345 | 3922 | 3243 | 3772 | 3560 | 3795 | 4015 | 3969 | 4010 |
|  | *Rmax* | 0.021 | 0.028 | 0.023 | 0.028 | 0.024 | 0.025 | 0.024 | 0.022 | 0.023 | 0.022 |
|  | *R²* | 0.93 | 0.93 | 0.96 | 0.90 | 0.97 | 0.92 | 0.96 | 0.92 | 0.96 | 0.90 |
| **Intermediate phalanges** | *A* | - | - | 3.24 | 1.95 | 4.35 | 2.58 | 4.00 | 2.53 | 2.84 | 1.83 |
|  |  | - | - | 3.08 | 1.86 | 4.17 | 2.46 | 3.82 | 2.38 | 2.71 | 1.69 |
|  |  | - | - | 3.39 | 2.05 | 4.53 | 2.71 | 4.17 | 2.67 | 2.98 | 1.96 |
|  | *b* | - | - | 0.95 | 1.00 | 0.98 | 1.01 | 0.94 | 0.96 | 0.97 | 0.97 |
|  |  | - | - | 0.87 | 0.91 | 0.89 | 0.90 | 0.87 | 0.87 | 0.87 | 0.89 |
|  |  | - | - | 1.04 | 1.10 | 1.07 | 1.13 | 1.02 | 1.04 | 1.08 | 1.04 |
|  | *k* | - | - | 0.0007 | 0.0007 | 0.0007 | 0.0008 | 0.0007 | 0.0007 | 0.0007 | 0.0006 |
|  |  | - | - | 0.0006 | 0.0006 | 0.0006 | 0.0006 | 0.0006 | 0.0005 | 0.0006 | 0.0005 |
|  |  | - | - | 0.0009 | 0.0008 | 0.0009 | 0.0009 | 0.0009 | 0.0009 | 0.0009 | 0.0007 |
|  | *Tf* | - | - | 3480 | 3612 | 3350 | 3375 | 3360 | 3551 | 3469 | 4177 |
|  | *Rmax* | - | - | 0.026 | 0.026 | 0.028 | 0.028 | 0.027 | 0.026 | 0.027 | 0.022 |
|  | *R²* | - | - | 0.93 | 0.88 | 0.94 | 0.89 | 0.94 | 0.84 | 0.91 | 0.84 |
| *For parameters *A*, *b* and *k,* the first row provides the average estimate, while the second and third rows provide the lower and upper 95% confidence intervals, respectively. | | | | | | | | | | | |

**Figure A.** Allometric relationships between hand and foot lengths and body mass in growing male and female *P. troglodytes* per individual (n = 15)*.* Data are plotted on a log-log scale and the allometry for the full group is quantified using A: linear mixed-effects (LME; black line) and B: Reduced Major Axis (RMA; blue line) regression models. The red dashed line in each panel indicates isometric scaling (i.e., a scaling exponent of 0.333).

**Individual: Alf**

**Individual: Art**

**Individual: Banka**

**Individual: Bard**

**Individual: Falla**

**Individual: Fanny**

**Individual: Flora**

**Individual: Jed**

**Individual: Jenny**

**Individual: Jent**

**Individual: Jojo**

**Individual: Karla**

**Individual: Ken**

**Individual: Scarf**

**Individual: Web**
